# Food Insecurity, Health and Nutritional Status among Sample of Palm-plantation Households in Malaysia

**DOI:** 10.3329/jhpn.v30i3.12292

**Published:** 2012-09

**Authors:** M. Mohamadpour, Z. Mohd Sharif, M. Avakh Keysami

**Affiliations:** ^1^Department of Research Management, Bushehr University of Medical Sciences, Islamic Republic of Iran; ^2^Faculty of Medicine and Health Sciences, Department of Community Nutrition, Universiti Putra Malaysia; ^3^Technical and Vocational Higher Education Center of Jihad-Agriculture Ministry, Bushehr, Islamic Republic of Iran

**Keywords:** Diet diversity, Food insecurity, Health risks, Nutritional status, Malaysia

## Abstract

Food insecurity is a worldwide problem and has been shown to contribute to poor health and nutritional outcomes. In Malaysia, poor dietary intake, overweight and obesity, diabetes mellitus, and hypercholesterolaemia have been reported to be more prevalent in females compared to males and in Indians compared to other ethnic groups. A cross-sectional study was designed to investigate the relationship between food insecurity and health and nutritional status among 169 Indian women (19-49 years old, non-pregnant, and non-lactating) from randomly-selected palm-plantation households in Negeri Sembilan, Malaysia. Subjects were interviewed for socioeconomic and demographic data, and information on household food security and dietary intake. They were examined for weight, height, waist-circumference, blood pressure and lipids, and plasma glucose levels. For analysis of data, descriptive statistics, ANOVA, and logistic regression were used. Majority (85.2%) of the households showed food insecurity as assessed using the Radimer/Cornell Hunger and Food Insecurity Instrument. The food-secure women had significantly higher mean years of education and lower mean number of children than food-insecure groups (p<0.05). There was a significant decrease in the mean household income and income per capita as food insecurity worsened (p<0.05). Women who reported food security had significantly higher mean diet diversity score (11.60±4.13) than child hunger (9.23±3.36). The group of subjects with higher intake of meat/fish/poultry/legumes (crude odds ratio [OR]=0.53, confidence interval [CI]=0.29-0.95) and higher diet diversity score (crude OR=0.87, CI=0.78-0.97) was more likely to have <3 health risks. Diet diversity score remained a significant protective factor against heath risks even after adjusting for other variables. The present study showed that food insecurity is indirectly associated with poor health and nutritional status. Therefore, appropriate community-based interventions should be designed and implemented to address the problems of food insecurity and possible health and nutritional outcomes.

## INTRODUCTION

Food insecurity is a worldwide problem, and it is a common phenomenon among the poor households in many developed and developing countries and has been shown to contribute to poor health and nutritional outcomes. Food insecurity, as explained by the Life Sciences Research Office, implies a condition “whenever the availability to nutritionally adequate and safe foods or the ability to acquire acceptable foods in socially acceptable ways is limited or uncertain” (1,2). The most severe form of food insecurity prevails in households where children are experiencing reduced food intake and hunger (3).

Food insecurity can affect health either directly or indirectly through nutritional status as indicated by undernutrition or overnutrition (4). Food insecurity is related to lower macro- and micronutrient intakes, lower intake of fruits and vegetables, and lack of diet diversity. These items contribute to higher prevalence of underweight or overweight and obesity, higher or lower blood cholesterol levels, lower serum albumin, lower haemoglobin, and vitamin A, disordered eating behaviours, and adverse physical and mental health (5-7). In Malaysia, there are differences in health and nutritional status by gender and ethnic groups. Poor dietary intake, overweight and obesity, diabetes mellitus, and hypercholesterolaemia have been reported to be more prevalent in females compared to males and in Indians compared to other ethnic groups (8-10). There are many factors associated with poor health and nutrition among women and Indians in Malaysia, and one is poverty. Poverty is one of the risk factors for food insecurity in that food-insecure individuals tend to have lower quantity and quality of food intake. Many studies in developed and developing countries have shown that food insecurity is associated with poor health and nutritional status (6,7,11). Although the relationship between poverty and poor health and nutrition is well-established in Malaysia (12), not much is known on the relationship between food security and poor health and nutritional status among Indian women in palm-plantation households, and this study was designed to investigate this association.

## MATERIALS AND METHODS

The study was conducted on randomly-selected palm-plantation households in Negeri Sembilan, Malaysia, during March–August 2007 to evaluate the relationship between food insecurity and health and nutritional status. The subjects were Indian women (19-49 years old, non-pregnant, and non-lactating). Since they were in the reproductive age-group, we found sufficient children in these households to see some level of food insecurity among children, popularly called ‘child hunger’.

The number of households needed for this study was calculated based on the prevalence of food insecurity (58%) among rural households in Sabak Bernam (11). The calculation of sample-size was based on method described by Chochran (13). The minimum calculated sample-size was 94. A list of all palm plantations (11) under the Guthrie Group (a central office for Malaysia Palm Plantation Management Company Guthrie Berhad) was obtained from the Guthrie Group Headquarters (Kumpulan Guthrie Berhad). The seven palm plantations, namely Bukit Pelandok, Ladang Labu, Ladang P.D., Lukut Siliau, Tampin Linggi, Sengkang, and Tanah Merah, were randomly selected. There were 377 Indian households in these seven palm plantations, and 169 women gave their consent to participate. All of 169 women were included in this study. During blood collection, 22 respondents refused to have their blood drawn.

The Ethics Committee of the Faculty of Medicine and Health Sciences, Universiti Putra Malaysia, approved the study protocol. The permission to conduct the study on palm plantations in Negeri Sembilan was also obtained from the Guthrie Group Headquarters and estate managers.

Interviews were conducted in the homes of the women by four trained Indian enumerators since most of the women could not speak Malay language very well. It was administered to women only in the absence of the household heads, such as the father, mother-in-law, or other family members. This was done to prevent any concealment of the actual food situation in the household by the women or embarrassment in responding to the items in the presence of other family members. Households’ sociodemographic and economic information was obtained from the women, using a structured questionnaire. The Radimer/Cornell Hunger and Food Insecurity Instrument (10 items) was used for determining the severity of food insecurity in a household (Table 1). These items were developed from the perspectives of rural women in upstate New York, who had experienced hunger. Further studies were conducted to establish the validity and reliability of the instrument (14,15). These 10 items were translated into Malay language, and the translated version was used in previous local studies (11,16,17). The internal consistency of the 10 items (Cronbach's alpha) in these studies were in the range of 0.8-0.9. The internal consistency of these 10 items (Cronbach's alpha) in the present study was 0.93. The instrument classifies the households as food-secure, household food insecurity, individual food insecurity, and child hunger.

Weight, height, and waist-circumference were measured using Tanita weighing scale and Seca body meter (18). Each weight, height, and waist-circumference was measured twice for each subject, and the average of the two readings was taken as final.

Food intake information was obtained through a 24-hour dietary recall (2 days) and food-frequency questionnaire (FFQ). The women were asked to recall the food and beverages consumed in the previous 24-hours and to estimate the portion-sizes, using a set of calibrated household measurements (e.g. by spoons, cups, glasses, bowls, plates, saucer, and ladles). The dietary intake data were then transformed into energy and nutrient intakes based on the Malaysian food database, using Nutritionist Pro software (First Data Bank). Energy (kcal), and nutrient intakes were evaluated for adequacy, using the Malaysian Recommended Nutrient Intake (19). The Malaysian Food Guide Pyramid was used as the basis to calculate the number of servings from each food group (20,21). The FFQ consisted of 29 food groups that were commonly consumed in Malaysia. There are 5 categories of grains and cereals, 4 categories of meat and meat products, 3 categories of fish and sea-foods, 5 categories of fruits, 5 categories of vegetables, 3 categories of milk and dairy products, 2 categories of meat-alternatives, and 2 categories of beverages. Diet diversity score (DDS) was calculated based on the consumption of these 29 food groups. The diet diversity score for each woman was calculated as food groups consumed daily or ≥2 times per week (22-24). The maximum DDS was 29. The score was zero for food group consumed less than 2 times per week or less than 8 times per month or never.

**Table. 1.** The Radimer/Cornell Hunger and Food Insecurity Items

Household Hunger:
Food anxiety component
1. I worry whether my food will run out before I get money to buy more.
Quantitative component
2. The food that I bought just didn't last, and I didn't have money to get more.
3. I ran out of foods that I needed to put together a meal and I didn't have money to get more food.
Qualitative component
4. We eat the same thing for several days in a row because we only have a few different kinds of food at hand and don't have money to buy more
Women's Hunger:
Qualitative component
5. I can't afford to eat properly
Quantitative component
6. I am often hungry, but I don't eat because I can't afford enough food
7. I eat less than I think I should because I don't have enough money for food
Children's Hunger:
Qualitative component
8. I can't give my child(ren) a balanced meal because I can't afford that
Quantitative component
9. My child(ren) is/are not eating enough because I just can't afford enough food
10. I know my child(ren) is/are hungry sometimes, but I just can't afford more food
**To classify individuals by severity of food insecurity**
Food-secure: negative answers to all hunger and food insecurity items
Household Insecurity: positive answers (sometimes true or often true) to one or more household-level item(s) (1-4) but not to adult or child-level items
Individual Insecurity: positive answers to one or more adult-level item(s) (5-7) or the item about the quality of children's diet (8), but not to items about the quantity of children's intake (9-10)
Child Hunger: positive answers to items about the quantity of children's intake (9-10)
Negative answer: ‘not true’ and Positive answer: ‘sometimes true’ or ‘often true’

Figures in parentheses are serial numbers of the above responses

Blood pressure was measured using the Omron digital blood pressure machine (Model MX3) and classified according to the World Health Organization/International Society of Hypertension Guidelines (25). The women were requested to fast overnight (8-10 hours) before blood collection. The blood samples were centrifuged using KUBOTA machine (Model 2100) for 15 minutes at 3000 rpm to obtain serum for lipids [total cholesterol, triglyceride, low-density lipoprotein (LDL)-cholesterol, high-density lipoprotein (HDL)-cholesterol], and plasma for fasting plasma glucose test. The blood samples were then analyzed using Chemical Analyzer Machine (HITACHI 902). The classifications of blood serum are based on the National Cholesterol Education Program (NCEP) guidelines (26). A normal fasting plasma glucose (FPG) is defined as <110 mg/dL or 6.1 mmol/L (27).

The National Cholesterol Education Program Adult Treatment Panel III (NCEP ATPIII) recommended the use of five variables for the diagnosis of metabolic syndrome. The measurement of the health risks is based on NCEP ATPIII. The measurements include waist-circumference (WC), serum triglyceride, serum HDL-C, blood pressure, and fasting plasma glucose. In this study, individuals with three or more risks were classified as having health risks (28).

### Statistical analysis

Data were analyzed using the Statistical Package for Social Science (SPSS) for Windows version 16.0 (SPSS Inc., Chicago, IL, USA). The first step in the analysis was to compare socioeconomic and demographic variables, anthropometric measurements, energy and nutrient intakes, diet diversity score, number of servings from each food group, blood pressure, lipid, and glucose levels according to food insecurity status. One-way analysis of variance (ANOVA) for continuous variables and chi-square for categorical variables were used. Post-hoc Bonferroni test was used for determining significant differences of the outcome variables among the food insecurity groups (food-secure, household food insecurity, individual food insecurity, and child hunger). All data were normally distributed. Logistic regressions were used for determining factors associated with 3 or more health risks relating to metabolic syndrome, such as at-risk waist-circumference, high blood pressure, hypertriglyceridaemia, low HDL-C, and high glucose level. All covariates were continuous, except for employment status (working or housewifery), blood pressure (normal, high), and food security status (food-secure and food-insecure). The results of logistic regressions were expressed as both crude and adjusted odds ratios (ORs), with 95% confidence intervals (CIs). The odds ratio greater than 1.00 is a risk factor for health while lower than 1.00 has a protective effect on health risks.

**Table. 2.** Socioeconomic and demographic characteristics of women by food security status (N=169)

Measurement	Food-secure (n=25)	Household food insecurity (n= 42)	Individual food insecurity (n=33)	Child hunger (n=69)	p value1
Age (years)					
Mean±SD	37.12±9.09	39.52±7.30	40.61±7.37	38.74±6.77	0.330
Years of schooling					
Mean±SD	6.60±3.39**, ***	6.17±3.21****, ******	4.64±3.38	4.57±3.31	0.011
Household-size					
Mean±SD	5.28±2.19	6.26±1.90	6.27±1.85	6.39±1.88	0.097
Number of children					
Mean±SD	2.64±1.58*, **, ***	3.43±1.66****	3.48±1.41	4.06±1.48	0.001
Household income					
Mean±SD	1229.73±672.59*, **, ***	953.95±482.045****	881.03±428.07*****	645.84±320.69	0.000
Income per capita					
Mean±SD	261.26±164.47 *, **, ***	177.51±130.96****	148.39±72.40	112.51±72.83	0.000

1One-way ANOVA;

*Significantly different between food-secure and household food insecurity;

**Significantly different between food-secure and individual food insecurity;

***Significantly different between food-secure and child hunger;

****Significantly different between household food insecurity and child hunger;

*****Significantly different between individual food insecurity and child hunger;

******Significantly different between household food insecurity and individual food insecurity;

all values are mean±SD;

SD=Standard deviation

## RESULTS

Overall, majority (85.2%) of the households showed food insecurity. Among the food-insecure groups, 24.9% experienced household food insecurity, 19.5% experienced individual food insecurity, and 40.8% experienced child hunger. As shown in Table 2, there was a decreasing trend in the mean years of education and increasing trend in the mean number of children with severity of food insecurity (p<0.05). There was a significant decrease in the mean household income and income per capita as food insecurity worsened (p<0.05). For dietary intake, women from food-secure households (469.18±254.52) and those with individual food insecurity (482.71±289.70) had significantly higher mean intake of vitamin A than those experiencing child hunger (327.66±183.08). Table 3 shows that food-secure women had significantly higher mean diet diversity score than women experiencing child hunger (p<0.05). Among the four food groups, food-secure women consumed significantly higher mean number of servings from protein group than women experiencing child hunger.

Table 4 shows that women from all food-insecure households had higher mean body mass index and higher prevalence of at-risk body mass index (BMI ≥25). However, the differences and association were not statistically significant. The percentage of women with waist-circumference ≥88 cm was the highest in child hunger (76.2%), followed by household insecurity (69.0%), food-secure (64.0%), and individual food insecurity groups (54.5%) (p<0.05). There were no significant mean differences in total cholesterol, triglycerides, HDL-cholesterol, LDL-cholesterol, plasma glucose, and blood pressure by food security status.

The health risks are referred to as waist-circumference ≥88 cm, serum triglycerides ≥150 mg/dL or ≥1.7 mmol/L, serum HDL-C <50 mg/dL or <1.29 mmol/L, blood pressure (systolic ≥130 and diastolic ≥85), and fasting glucose ≥110 mg/dL or ≥6.1 mmol/L. These cut-off points are based on The National Cholesterol Education Program Adult Treatment Panel III for female (28). Women with three or more of these health risks can be classified as at risk of metabolic syndrome. The mean number of health risks was significantly higher in child hunger (2.27±1.20) compared to individual food insecurity group (1.48±1.05). Although the percentage of women with ≥3 health risks was the highest in child hunger group (42.9%) and the lowest in food-secure group (17.6%), there was no significant association in the distribution of health risks by food security status (Table 5).

**Table. 3.** Diet diversity score and food group intake (number of servings) of women by food security status (N=169)

Measurement	Food-secure (n=25)	Household food insecurity (n=42)	Individual food insecurity (n=33)	Child hunger (n=69)	p value^1^
Diet diversity score					
Mean±SD	11.60±4.13*	10.31±3.21	10.52±3.47	9.23±3.36	0.025
Food group (no. of servings):					
Grains/cereals/tubers					
Mean±SD	6.56±1.33	6.39±1.62	6.24±1.63	6.19±1.77	0.781
Fruits/vegetables					
Mean±SD	1.27±0.75	1.26±0.59	1.43±0.78	1.28±0.83	0.762
Meat/fish/poultry/legumes					
Mean±SD	1.44±0.73*	1.30±0.59	1.16±0.57	1.06±0.60	0.042
Milk/dairy products					
Mean±SD	0.30±0.51	0.39±0.46	0.37±0.45	0.25±0.43	0.407

1One-way ANOVA;

*Significantly different between food-secure and child hunger group

**Table. 4.** Body mass index and waist-circumference of women by food security status (N=147)

Variable	Food-secure (n=17)	Household food insecurity (n=38)	Individual food insecurity (n=29)	Child hunger (n=63)	p value^a^ or chi-square value^b^
Body mass index (kg/m2)					
Mean±SD	25.64±4.60	26.59±5.73	26.49±4.30	27.06±4.48	0.744
BMI <25.00	9 (52.9)	14 (36.8)	9 (31.0)	20 (31.7)	
BMI ≥25.00	8 (47.1)	24 (63.2)	20 (69.0)	43 (68.3)	χ2=2.932
Waist-circumference (cm)					
Mean±SD	88.01±10.72	92.13±13.26	89.83±9.97	93.27±10.27	0.264
<88 cm (35 inch)	8 (47.1)	12 (31.6)	15 (51.7)	15 (23.8)	
≥88 cm (35 inch)	9 (52.9)	26 (68.4)	14 (48.3)	48 (76.2)	χ2=8.365*

^a^One-way ANOVA;

^b^Chi-square value is based on BMI <25 (non-overweight/obese) and BMI ≥25 (overweight/obese); WC <88 cm and WC ≥88 cm;

*p<0.05; Figures in parentheses are percentages;

WC=Waist-circumference

Table 6 presents the crude and adjusted odds ratio with 95% confidence intervals for women with ≥3 health risks. In the crude odds ratio model, women with higher intake of meat/fish/poultry/legumes (crude OR=0.53, CI=0.29-0.95) group and higher diet diversity score (crude OR=0.87, CI=0.78-0.97) were more likely to have <3 health risks. Diet diversity score (adjusted OR=0.87, CI=0.76-0.99) remained a significant protective factor against health risks even after adjusting for other variables.

**Table. 5.** Health risks of women by food security status (N=147)

Category	Food secure (n=25)	Household food insecurity (n=42)	Individual food insecurity (n=33)	Child hunger (n=69)	p value^a^ or chi-square value^a^
Criteria for health risks					
Mean±SD	1.76±0.07	1.95±1.23	1.48±1.05*	2.27±1.20	0.026
Women with <3 risk factors	14 (82.4)	28 (73.7)	23 (79.3)	36 (57.1)	
Women with ≥3 risk factors	3 (17.6)	10 (26.3)	6 (20.7)	27 (42.9)	χ2=7.345

^a^One-way ANOVA;

^b^Chi-square test;

*Significantly different between individual food insecurity and child hunger group;

Figures in parentheses are percentages

**Table. 6.** Crude and adjusted odds ratio and 95% confidence intervals for women with ≥3 heath risks^a^ (N=147)

Variable	Crude OR	(95% CI)	p value	Adjusted OR^b^	(95% CI)	p value
Age (years)	1.04	(0.99-1.09)	0.157	1.06	(0.99-1.13)	0.084
Years of education	0.97	(0.87-1.08)	0.548	1.04	(0.92-1.19)	0.532
Household-size	1.08	(0.90-1.29)	0.426	1.03	(0.77-1.39)	0.835
Number of children	1.12	(0.90-1.39)	0.298	1.04	(0.72-1.51)	0.837
Employment						
Worker	1.00			1.00		
Housewife	1.71	(0.80-3.66)	0.166	2.33	(0.83-6.55)	0.108
Total income	1.00	(1.00-1.00)	0.252	1.00	(1.00-1.00)	0.962
Energy	1.00	(1.00-1.00)	0.338	1.01	(0.99-1.02)	0.465
Carbohydrate	1.00	(0.99-1.00)	0.222	0.98	(0.92-1.04)	0.441
Total fat	1.00	(0.98-1.02)	0.981	0.96	(0.85-1.09)	0.560
Vitamin A	1.00	(1.00-1.00)	0.731	1.00	(1.00-1.00)	0.685
Vitamin C	1.00	(0.99-1.02)	0.652	1.00	(0.98-1.03)	0.843
Diet diversity score	0.87*	(0.78-0.97)	0.010	0.87*	(0.76-0.99)	0.029
Food group (no of servings)						
Grains/cereals/tubers	0.82	(0.65-1.02)	0.077	0.86	(0.59-1.25)	0.434
Fruits/vegetables	1.28	(0.82-2.01)	0.282	1.35	(0.65-2.82)	0.425
Meat/fish/poultry	0.53*	(0.29-0.95)	0.034	0.44	(0.17-1.12)	0.084
Milk/dairy products	0.45	(0.18-1.12)	0.084	0.42	(0.15-1.20)	0.105
Food security status						
Food-secure	1.00			1.00		
Food-insecure	2.31	(0.63-8.46)	0.207	1.94	(0.41-9.11)	0.401

*p<0.05;

^a^(≥3 risk factor=presence of health risk and <3 risk factor=absence of health risk);

^b^Adjusted for age, year of education, household-size, number of children, occupation of women, total income, energy, carbohydrate, total fat, vitamin A, vitamin C, diet diversity score, number of servings from each food group (grains/cereals/tubers, fruits/vegetables, meat/fish/poultry/legumes and milk/dairy products), and food security status

## DISCUSSION

In the present study, a majority (85.2%) of the households experienced some form of food insecurity, namely household food insecurity (24.9%), individual food insecurity (19.5%), and child hunger (40.8%). The prevalence of food insecurity in the present study was higher than those reported in several local studies in Malaysia (11,16,17).

The findings of this study on child hunger distribution were similar to the findings in the rural area of Sabak Bernam, which reported that the prevalence of child hunger (34.5%) was also the highest among the food-insecure groups (11). Conversely, Zalilah and Ang reported that, among low-income households in Kuala Lumpur, the most experienced level of food insecurity was the household food insecurity (27.7%) (16). Zalilah and Tham also found that the individual food insecurity (32.8%) was most-commonly experienced by the Orang Asli households in Hulu Langat (17). Among the rural New York residents, the most experienced level of food insecurity was the household food insecurity (25%) while in Korea, it was the individual food insecurity (28.4%) (15,29). The reason for the higher percentage of individual and household food insecurity than the percentage of child hunger in Korea and rural New York was not mentioned.

The reasons for the higher percentage of households with child hunger than household or individual food insecurity in the present study could be related to under-reporting of household and individual food insecurity and over-reporting of child hunger. Under-reporting of household food insecurity may be due to perception of mother about adequacy of food in the households. The prevalence of individual (mother's) food insecurity may be under-reported because mothers may not report the truth as they may be embarrassed to answer these questions that are directly related to them. Thus, it is easier for mothers to respond to the questions relating to child hunger (16). Also, the mothers may have the perception that food supply in the households is not adequate for children's growth, and this may lead to over-reporting of child hunger. Finally, the reported prevalence of child hunger among the Indians could be a true prevalence as many of the households with child hunger (62.3%) had an income of ≤US$ 28 per capita.

The findings on household income are supported by several studies in developing and developed countries that found income as an important determinant of household food insecurity (30-33). Among the poor households, inadequate income can contribute to inability to provide sufficient foods for the household members. In the present study, the mean years of schooling significantly decreased as food insecurity worsened, which is reported by several studies. The improvement in education leads to better opportunities for employment, which eventually can improve household food security levels (30-33). The dietary intake data showed that a high percentage of women had intakes of energy, and most of the nutrients were below the recommended levels. A study by Ismail also reported that females of all ethnic groups in Malaysia had energy intake below the Malaysian RDA (34). The mean energy intake of Indian women in the present study (1,193 kcal) was similar to that (1,224 kcal) reported on women in the urban squatters of Malaysia (35). A study on estate workers showed higher energy (1,538 kcal) but similar protein intakes compared to the subjects in the present study (36). The protein intake by women in this study was less than those reported in other studies (35,37). The reasons for differences in the findings of these studies could be the difference in sample-size, subjects, ethnicity, location of study, and different methodologies for measuring dietary intakes. The present study was conducted in a sample with a high prevalence of food insecurity in which the role of income can be a very important determinant of dietary intakes. The food-insecure households may purchase food with lower quality or even lower quantity due to limited income. Living in the palm plantations may also affect food availability and accessibility in that the households have limited access to markets, lack of land for subsistence farming and payment of higher costs for food. The low mean energy and nutrient intakes among the women in this study could also be due to under-reporting of food intakes as the mean ratio of energy intake to basal metabolic rate (EI:BMR) was 0.9. The mean energy and nutrient intakes could also reflect the actual intakes of these women due to food insecurity.

In the present study, the mean number of servings from the four food groups was less than the recommended servings based on the Malaysian Food Guide Pyramid. The mean intake of vitamin A, diet diversity score, and number of servings from meat/fish/poultry/legumes decreased significantly as food insecurity worsened (p<0.05). Several studies have reported that food-insecure households have lower nutrient intakes, diet diversity or variety, and number of servings from expensive food groups (meat/fish/poultry/legumes and fruits and vegetables) compared to food-secure households (5,38-40). All of these studies found that the experience of food insecurity is likely to affect the quality, quantity, and eating behaviours. In the present study, the women not only had reduced energy and nutrient intakes but also reduced quality of food intakes (lower intake of meat, fruits, and vegetables). Except vitamin A, the intake of other nutrients did not show any significant difference by food security levels. There are studies that reported lower intakes of iron, calcium, magnesium, vitamin A, B_6_, C, E, folate, and zinc among food-insecure than food-secure women (41,42). Homogeneity of the study samples may be a possible explanation for lack of significant difference in most nutrients by food security levels. As both food-secure and food-insecure women are living in palm plantations and belong to the same ethnic groups, they may have access to similar foods or share similar food culture.

The anthropometric measurements indicated that many women were not only overweight (39.1%) and obese (26.0%) but also at risk of having abdominal adiposity (68.6%). Several studies in Malaysia have also reported high prevalence of overweight, obesity, and at-risk waist-circumference compared to normal weight and underweight in females and Indian populations (9,35,37). A possible reason for high prevalence of obesity in Indian females is related to low diet quality (higher consumption of refined grains, foods of animal source, sugar, and fat and lower intake of fruits and vegetables). Most Indian women in palm plantations (97.6%) consumed less than the minimum recommended number of servings (3 servings) of fruits and vegetables. Contribution of fat to total energy in more than one-third of these women was ≥30%. Therefore, lower quality of diet (higher intake of fat and lower intake of fruits and vegetables), with moderate to high physical activity, may also contribute to accumulation of body fat, consequently leading to high prevalence of overweight, obesity, and at-risk waist-circumference (9,35,37).

The current study did not find association between obesity and food security status. Similarly, Guilliford *et al.* reported that food insecurity in Trinidad was prevalent in all BMI groups and was significantly associated with underweight but not overweight (5). However, based on chi-square test, a significant association was observed between at-risk waist-circumference and food security in the present study. A study in Malaysia also reported that more women in food-insecure groups (32-47%) than those in the food-secure group (29%) had at-risk wait-circumference (11).

There are several reasons that may explain the higher prevalence of at-risk BMI and waist-circumference in the food-insecure women than food-secure group. First, consuming cheap foods that are typically lower in nutritional quality and contain higher levels of calories per dollar (lower intake of fruits and vegetables, milk and dairy products, added fat, sugar and higher intake of rice) may contribute to overweight, obesity, and abdominal adiposity (43,44). Second, food deprivation in low-income households promotes binge eating (overeating when food is available and restriction at the time of food insufficiency) and contributes to cycling of weight gain and weight loss, which could contribute to development of overweight and obesity (45,46). Third, it is possible that other factors, such as genetics (family history of obesity), behaviour of food intake (added sugar or fat), environment (physical activity), and socioeconomic conditions (education, income), contribute to obesity and abdominal adiposity (47-49).

There was no significant mean difference and association between glucose and lipid profiles by food security levels. To date, there have been few studies linking food insecurity to biochemical measures of nutritional status among women. Dixon *et al*. (42) reported that women from food-insecure families had lower serum concentration of HDL-cholesterol compared to food-secure group. A study by Stuff *et al.* on 1,453 households in the Lower Mississippi Delta reported that adults in food-insecure than food-secure households were significantly more likely to report presence of high cholesterol (20.6 vs 17.9%) and high blood glucose (15.1 vs 4%) (6). In the present study, majority (80.3%) of the women had normal blood pressure (<130/85 mmHg), and 19.7% (nearly one-fifth of the women) had high blood pressure (≥130/85 mmHg). The prevalence of hypertension in this study is higher compared to the report of the First Health and Morbidity Survey (NHMSI) in 1986 (14.4%) but lower compared to the report of the NHMS II (29.9%). Yunus *et al.* also reported high prevalence of hypertension (26.8%) among adults of all ethnic groups (Malays, Chinese, Indian, and Orang Asli) in Mukim Dengkil (50). However, the differences in prevalence may be related to different ethnicity of the subjects and higher sample-size (n=570) in the study by Yunus *et al.* compared to the present study (n=147). A study by Nicholas and Tarasuk showed a significantly higher mean blood pressure among adults in food-insecure households compared to food-secure households (51). In the present study, there was an increasing trend in the distribution of women with at-risk blood pressure with severity of food insecurity from 5.9% in food-secure to 25.4% in child hunger. However, the association was not statistically significant. Homogeneity of the sample and small sample-size may contribute to lack of significant mean difference and association of blood glucose and lipid by food security levels.

The high mean number of health risks and distribution of women with ≥3 health risks in food-insecure groups may be related to two factors. First, the food-insecure groups had lower mean income compared to food-secure groups, and the higher cost of medication and medical bills may put them at more risk of poor health (51,52). Second, stress and anxiety due to lack of food for household members may produce hormonal imbalances and high blood pressure, and these together may increase weight gain, obesity, insulin insensitivity, and other health problems (53).

Higher intake of foods from protein group was significantly protective against having ≥3 health risks and low HDL-cholesterol. This protective effect could be due to the use of fish and legumes as major sources of protein by Indian women. The 24-hour dietary recall showed that the mean number of servings from meat/fish/poultry/legumes food group among women is less than the recommended value (1-2 times per week). However, according to FFQ, more than 70% of women had at least two fish meals each week. The main types of fish consumed were sardines, herring, and Indian mackerel. These fishes are high in omega-3, a fatty acid that could increase HDL-cholesterol and decrease triglyceride (54,55). Bulliyya reported that the mean values of serum LDL-cholesterol and total cholesterol were significantly lower and HDL-cholesterol was higher in fish-consuming individuals than those who did not consume fish (20-70 years old) in India (56). Several studies have reported that the consumption of legumes may decrease the risks of metabolic syndrome, cardiovascular disease, hypertension, high plasma total cholesterol, triglyceride, LDL-cholesterol, and low HDL-cholesterol (57, 58). Indian women with higher diet diversity score (adjusted OR=0.87, CI=0.76-0.99) were significantly less likely to have ≥3 health risks. Several studies have also shown that diet diversity was associated with health status (59-61). A longitudinal (14 years) study of 10,337 men and women, with 988 having cardiovascular diseases, 571 cancer, and 910 other cases, found that mortality from cardiovascular disease and other cases (except cancer) was inversely related with diet diversity score in both men and women (59). A study of 1,993 cases and 2,140 controls in the United States found that women who consumed a diet with a high diversity of vegetables were at 20% lower risk for colon cancer than women who consumed a diet with no diversity of vegetables (60). La Vecchia *et al.* also reported that those with a higher diet diversity are protected against gastric cancer (61).

Finally, the findings of the present study that the food insecurity was not a significant risk factor for any of the health risks is in contrast to the findings of several studies (6,51,62). In the present study, food insecurity was found associated with dietary intake (diet diversity and number of servings from meat/fish/poultry/legumes food group), waist-circumference and number of health risks. Based on conceptual framework in the study, perhaps the effect of food insecurity on health risks is exerted through dietary intakes, which may have impact on waist-circumference and, consequently, on other health risks. The diet diversity may be a useful indicator of household food security; several studies showed that food insecurity is associated with low diet diversity (40,63-65). As households become food-insecure, the ability to obtain variety of foods will be compromised and consequently will put the women at risk of having bigger waist-circumference and health problems.

### Limitations

The dietary data were collected using 24-hour dietary recall and food frequency questionnaire (FFQ). The accuracy of the data depended on the respondent's memory, honesty, and ability to understand the questions. To overcome these problems, the enumerators were trained to increase the ability in using the instrument. Data on 24-hour dietary recall and food frequency questionnaire were checked by researchers for accuracy, and those that appeared not correctly obtained were collected again through interview. It is also possible that women might not understand the 10 items of the Radimer/Cornell Hunger and Food Insecurity Instrument, had different interpretation of the items or might be embarrassed to give answers to the questions. These problems could contribute to under- or overestimation of the prevalence of household food insecurity. However, to overcome these problems, the items were translated into Tamil language, and Indian enumerators were trained to administer the items. The women were interviewed in absence of the husbands, parents or parents-in-law, or other family members to prevent any discomfort.

### Conclusions

In the present study, food insecurity was not a significant risk factor for any of the health risks but was associated with dietary intake and waist-circumference. Perhaps the effect of food insecurity on health risks is exerted through dietary intakes, which may have impact on waist-circumference. Therefore, the present study showed that food insecurity among the Indian women from selected palm plantations in Malaysia is indirectly associated with poor health and nutritional status.

### Recommendation

The risk factors and consequences of household food insecurity among Indian women from selected palm plantations may be different from those in other ethnic groups and areas in Malaysia. Therefore, further studies in Malaysia need to be done in other ethnic groups and areas for an intervention programme to reduce poor health and nutritional outcomes of food insecurity.

## ACKNOWLEDGEMENTS

The project was funded by IRPA (Intensification of Research in Priority Areas) (Grant 54439 EAR), Faculty of Medicine and Health Sciences, Department of Community Nutrition, Universiti Putra Malaysia. The authors would like to thank Professor G.L. Khor, Professor M. Kandiah, and Associate Professor Y.M. Chan for their very useful comments during research. We would also like to thank Mr. Fakher Rahim from RCC of Ahvaz Jundishapur of Medical Sciences and the women who participated in this project.
